# Cerebrospinal fluid concentration of furin in patients with Alzheimer’s disease

**DOI:** 10.1002/alz.088780

**Published:** 2025-01-09

**Authors:** Agnieszka Kulczynska‐Przybik, Jan Mroczko, Maciej Dulewicz, Henrik Zetterberg, Jörg Hanrieder, Kaj Blennow, Daria Krawczuk, Julia Doroszkiewicz, Renata Borawska, Agnieszka Slowik, Barbara Mroczko

**Affiliations:** ^1^ Department of Neurodegeneration Diagnostics, Medical University of Bialystok, Bialystok Poland; ^2^ Department of Psychiatry and Neurochemistry, Institute of Neuroscience and Physiology, The Sahlgrenska Academy at the University of Gothenburg, Gothenburg, Gothenburg Sweden; ^3^ Department of Psychiatry and Neurochemistry, Institute of Neuroscience and Physiology, The Sahlgrenska Academy, University of Gothenburg, Mölndal, Gothenburg Sweden; ^4^ Department of Psychiatry and Neurochemistry, Institute of Neuroscience and Physiology, The Sahlgrenska Academy at the University of Gothenburg, Gothenburg Sweden; ^5^ Department of Psychiatry and Neurochemistry, Institute of Neuroscience and Physiology, The Sahlgrenska Academy, University of Gothenburg, Mölndal Sweden; ^6^ Department of Neurology, Jagiellonian University, Krakow Poland

## Abstract

**Background:**

Alzheimer's disease (AD) is an uncurable, heterogeneous, and molecular complex neurodegenerative disease. Emerging evidence indicates that furin could play an essential role in the pathogenesis of neurodegenerative disorders. Furin participates in the proteolytic maturation and processing of large numbers of prohormones and proproteins, which among others play crucial roles in neuronal survival, axon growth, dendritic development, synaptogenesis, neurodegeneration, and inflammation. It is suggested that a stable activity of furin is crucial for maintaining of the central nervous system homeostasis. Some studies revealed reduced expression of mRNA of furin in the brains of Alzheimer's disease patients, which may suggest the implication of the protein in the pathophysiology of AD. However, little is known about changes of furin’s concentration in the cerebrospinal fluid (CSF) patients with AD. Therefore, we aimed to assess the usefulness of CSF furin’s measurement in patients with AD in relation to subjects without cognitive impairment. We also assessed associations between CSF concentrations of furin and neurochemical biomarkers of dementia.

**Method:**

The study enrolled 39 participants, including patients with AD and subjects without cognitive decline. The concentrations of furin and classical biomarkers (amyloid beta 1‐42, amyloid beta 1‐40, Tau, as well as pTau181) were assessed in cerebrospinal fluid using a multiplexing method as well as enzyme‐linked immunosorbent assay.

**Result:**

A significantly higher CSF concentration of furin was noticed in AD patients as compared to subjects without cognitive impairment. Furthermore, in the whole study group of patients, the levels of furin correlated negatively with MMSE test and Aβ‐1‐42/ Aβ‐1‐40 ratio, as well as positively with total Tau and pTau181. Additionally, the CSF levels of furin significantly correlated with pTau181 protein in the AD group.

**Conclusion:**

Our preliminary study indicates the potential role of furin in the pathology of AD and its potential usefulness as a candidate biomarker. However, further investigations on an independent larger group of patients are necessary.

